# Microbial Surfactants: The Next Generation Multifunctional Biomolecules for Applications in the Petroleum Industry and Its Associated Environmental Remediation

**DOI:** 10.3390/microorganisms7110581

**Published:** 2019-11-19

**Authors:** Emmanuel O. Fenibo, Grace N. Ijoma, Ramganesh Selvarajan, Chioma B. Chikere

**Affiliations:** 1 World Bank Africa Centre of Excellence, Centre for Oilfield Chemical Research, University of Port Harcourt, Port Harcourt 500272, Nigeria; 2 Institute for the Development of Energy for African Sustainability, University of South Africa, Roodepoort 1709, South Africa; nkechiijoma@gmail.com; 3 Department of Environmental Science, University of South Africa, Florida Campus, Rooderpoort 1709, South Africa; 4 Department of Microbiology, Faculty of Science, University of Port Harcourt, Port Harcourt 500272, Nigeria; chioma.chikere@uniport.edu.ng

**Keywords:** biosurfactants, biotechnological applications, MEOR, synthetic surfactants, sustainability

## Abstract

Surfactants are a broad category of tensio-active biomolecules with multifunctional properties applications in diverse industrial sectors and processes. Surfactants are produced synthetically and biologically. The biologically derived surfactants (biosurfactants) are produced from microorganisms, with *Pseudomonas* *aeruginosa*, *Bacillus* *subtilis Candida albicans*, and *Acinetobacter* *calcoaceticus* as dominant species. Rhamnolipids, sophorolipids, mannosylerithritol lipids, surfactin, and emulsan are well known in terms of their biotechnological applications. Biosurfactants can compete with synthetic surfactants in terms of performance, with established advantages over synthetic ones, including eco-friendliness, biodegradability, low toxicity, and stability over a wide variability of environmental factors. However, at present, synthetic surfactants are a preferred option in different industrial applications because of their availability in commercial quantities, unlike biosurfactants. The usage of synthetic surfactants introduces new species of recalcitrant pollutants into the environment and leads to undesired results when a wrong selection of surfactants is made. Substituting synthetic surfactants with biosurfactants resolves these drawbacks, thus interest has been intensified in biosurfactant applications in a wide range of industries hitherto considered as experimental fields. This review, therefore, intends to offer an overview of diverse applications in which biosurfactants have been found to be useful, with emphases on petroleum biotechnology, environmental remediation, and the agriculture sector. The application of biosurfactants in these settings would lead to industrial growth and environmental sustainability.

## 1. Introduction

Surfactants are a class of chemical compounds possessing amphiphilic (both hydrophobic and hydrophilic) moieties that distribute themselves between two immiscible fluids, with the effect of reducing the surface/interfacial tensions and causing the solubility of polar compounds in non-polar solvents [1]. They display properties, such as detergency, solubilization, and lubrication; have stabilizing and foaming capacity; and form phase dispersion [2]. Surfactants are either derived synthetically or biologically. Naturally derived surfactants are denominated biosurfactants since they are produced from biological entities, especially microorganisms. Fungi, bacteria, and yeast belonging to different species and strains are known for producing biosurfactants of a diverse variety of molecular structures [3]. Amongst the bacteria domain, genera of *Pseudomonas*, *Bacillus*, and *Acinetobacter* dominate the literature space as excellent producers of biosurfactants [2]. The species among these genera that have been extensively studied are *Pseudomonas aeruginosa*, *Bacillus subtilis*, and *Acinetobacter calcoaceticus*, amongst other species [1,4,5]. Bhardwaj et al. [6] and Morita et al. [7] respectively studied *Candida bombicola* and *Pseudozyma rugulosa* representing fungi and yeast. Biosurfactants are broadly grouped into low molecular weight (LMW) and high molecular weight (HMW) biosurfactants based on their biochemical natures. The former efficiently lowers surface and interfacial tensions while the latter is more of an emulsion-stabilizing agent. On the basis of chemical composition, biosurfactants are grouped into glycolipids (rhamnolipids, sophorolipids, trehalolipids, mannosylerithritol lipids), lipopeptides (surfactin, lichenysin, iturin, fengycin, serrwettin), fatty acids/phospholipids/neutral lipids (phosphatidylethanolamine, spiculisporic acid), polymeric biosurfactants (emulsan, alasan, biodispesan, liposan), and particulate biosurfactants (vesicles, whole-cell) [8,9,10]. Lipopeptides, glycolipids, and phospholipids belong to the LMW biosurfactants while the HMW biosurfactants include polymeric and particulate biosurfactants [11]. Microbial and synthetic surfactants are employed in diverse industries, including the cosmetics, food, and pharmaceutical sector.

Surfactants have a versatile phase character and diversity of colloidal structures, thus finding application in many industrial processes, especially where modification of the interface activity or stability of the colloidal systems is required [12]. There are four categories of surfactants: Anionic, cationic, nonionic, and zwitterionic [13] based on the composition of the polarity of the head group. The anionic surfactants carry a negative charge, which is the most commonly available surfactants chemically and naturally [14,15]. They have prominent application in personal care products and soaps because they are very effective in cleansing systems [16]. Further, they are also used in the oil industry, agriculture, health, cosmetics, remediation, and bioprospecting because of their wide range of hydrophilic–hydrophobic balance (HLB) values, emulsification property, and their excellent ability to reduce surface tension. The positively charged surfactants (cationic) are well suited for surfaces with a negative charge, thus they are used as anti-corrosion/antistatic agents, flotation collectors, fabric softeners, hair conditioners, and bactericides [17]. The nonionics are surfactants with uncharged hydrophilic head groups, which are good in low-temperature detergents and emulsifiers probably because of their low irritating effects [18]. The zwitterionics are amphoteric surfactants with poor cleansing and emulsifying properties [14] but have excellent dermatological properties and skin compatibility [19]. Also, they are used in manufacturing shampoos and cosmetics. So, surfactants can be used in the petroleum industry, health, pharmaceuticals, agriculture, detergents, cosmetics, bioprocessing, environmental remediation, textiles, paint, leader, papermaking, and other industries and activities where water could serve as an interactive medium [16,20,21,22,23,24,25].

When synthetic surfactants are used to run industrial processes, two categories of pollutants are elicited: By-products from the industrial activity and the remnant of the surfactants. They are both hazardous to the environment and their receptors, including humans. These elicited chemicals are persistent in the environment because they are hardly biodegradable. Besides, they consume more energy in a system in which they are applied in comparison to biosurfactants [26]. With these disadvantages, it has become imperative to identify an alternative approach that is environmentally suitable without compromising performance. The use of biosurfactants in industries had proved that they are eco-friendly, cost-effective, biodegradable, biocompatible, easy to produce, have low toxicity, chemically diverse, and are stable against changes in environmental conditions. Despite their advantages over synthetic surfactants, biosurfactants’ output is comparatively low in the global market [27]. This limitation is a result of low productivity from microorganisms and the heavy cost attached to downstream processes. The high cost in downstream processes is associated with biosurfactants’ isolation and purification. The effects of these drawbacks can be reduced through the use of agricultural wastes as a carbon source, genetic modification of microorganisms, and growth optimization via computational modelling [3,10,27,28]. With the research interest devoted to the commercial production of biosurfactants, they will rapidly become a competitive substitute for synthetic surfactants, thus promoting the overall health and sustainability of the environment [28,29,30,31,32,33,34,35].

The application of biosurfactants in certain industrial sectors that are critical for sustainable economic development, especially in developing countries, remains a focus for every biotechnologist. Thus, this review attempts to offer an overview of the multifunctional properties of biosurfactants that influence their applications in current and diverse industrial sectors, with emphases on petroleum biotechnology, environmental remediation, and agriculture. Overall, the number of industries in which biosurfactants have found applications in prove that they have the potential to substitute synthetic surfactants in the nearest future.

## 2. Application of Biosurfactants in the Petroleum Industry

The observed demand for crude oil as a major energy source was 99.3 million barrels per day in 2018 and is projected to reach 100.6 million barrels per day in 2019 [36]. With this rate of consumption, medium and light oil will be exhausted, and a reliance will be placed on heavy and extra-heavy oils. Consequently, surfactants will be required to extract such heavy oils from reservoirs for global energy consumption. Currently, microbial surfactants have been employed in exploring heavy oil, with a record of comparative effectiveness without causing environmental impact because they are biodegradable, unlike synthetic surfactants. This advantage of eco-friendliness is what synthetic surfactants cannot offer in the entire chain of crude oil processing, exploration, transportation, and storage [37]. Also, biosurfactants are used in the formulations of emulsifying/demulsifying agents, anti-corrosives, biocides, and other innovative applications in the petroleum industry [38]. Biosurfactants had proved their usefulness in residual oil recovery by solubilizing trapped oil in rock formations, which is a prerequisite for enhanced oil recovery (EOR). By the same principle, they have also been used in washing contaminated vessels and facilitating pipeline transport of heavy crude oil [39]. Their anti-corrosion effects on oil-prospecting assets are based on the orientation of their polar groups and their antimicrobial activities [40].

### 2.1. Extraction of Crude Oil from Reservoirs

Oil production from wells is achieved through primary, secondary, and tertiary recovery methods. The primary and secondary methods employ natural pressure and induced pressure, respectively, to extract oil from the reservoir. The recovery of oil by these first two methods is calculated to be 40%, leaving 60% of the original oil trapped in the reservoir [41]. Further, to recover some of the trapped oil, tertiary recovery (enhanced oil recovery) is employed using thermal and non-thermal techniques. The non-thermal technique uses chemical flooding and biological methods. The latter is termed microbial enhanced oil recovery (MEOR). Alternatively, the chemical flooding technique (conventional method) boosts the pressure in the reservoir and also creates favorable conditions for trapped oil recovery [41]. These conditions include interfacial tension reduction between the oil and displacing fluid, viscosity reduction, reduction of capillary forces, increasing the drive–water viscosity, oil swelling, and alteration of the wettability of the reservoir rock [42,43]. The use of synthetic chemicals not only causes environmental pollution but is also capital intensive [44]. Besides, poor selection of synthetic surfactants can cause low oil recovery, undesirable wettability alteration, pore surface blockage, and rock dissolution through chemical reactions [45]. Biosurfactant applications in EOR can provide favorable conditions, which are highlighted [46], as well as resolve the disadvantages associated with environmental pollution and poor chemical selection. By simple definition, MEOR is an oil recovery technique in which microorganisms or their metabolic products are used to recover residual oil [47]. The procedure is usually accomplished by the injection of biosurfactant producers followed by nutrient injection into the reservoir or ex-situ production of biosurfactants and subsequent injection. The result is that microorganisms produce emulsifiers/surfactants that diminish the capillary forces inherent in the rock pore, reducing the oil–rock surface tension, and thereby resulting in the release of trapped oil [38,44,48]. In the presence of injected CO_2_, biosurfactants alter the gas wettability and CO_2_–brine–rock interfacial behavior, which improves the sweep efficiency of the injected fluid and displaced CO_2_ gas, hence resulting in oil recovery [49].

**Figure 1:**
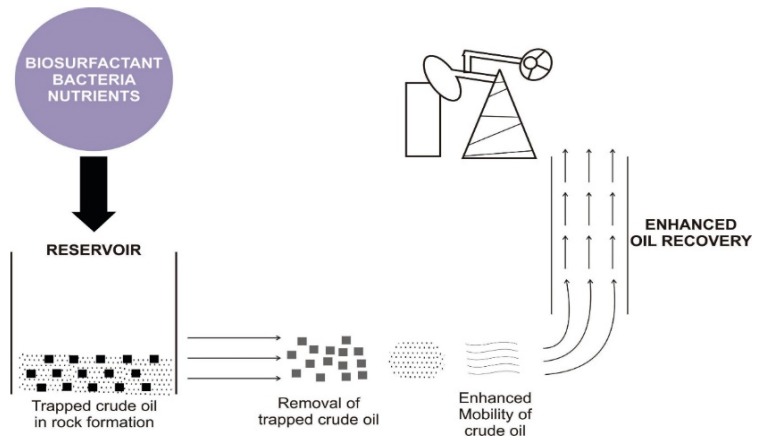
Working principle of biosurfactants in microbially enhanced oil recovery (MEOR).

The application of MEOR has led to the revival of reservoirs at a lower cost and minimal pollution in comparison to the use of the conventional EOR method [50]. More than 4600 oilfields used MEOR in China, with 500 wells operating via microbial flooding recovery [51]. A review conducted by Maudgalya et al. [52] reported the revival of 20 oil reservoirs out of 26 field trials. Khire [53] reported the use of an undisclosed biosurfactant (PIMP) to recover 11.2% of oil from a model reservoir and a decreased injection pressure by 40.1% while Golabi et al. used laboratory treatability studies (sand-pack column experiment) [54] to demonstrate a 15% oil recovery using crude biosurfactants. One of the outstanding successes of MEOR is the production of 9.75 × 10^4^ ton additional crude oil over a decade in the Shengli oil field, China [55]. Apart from being used in MEOR for residual oil recovery, they also inhibit corrosion that arises from the co-introduced air [38] and microbial activities. Biosurfactants interact with metal surfaces and orient the lipophobic head to the surface and the lipophilic tail to the external environment, thereby creating unfavorable conditions for corrosion. Furthermore, the antimicrobial effect of biosurfactants reduces the biomass of sulfate-reducing bacteria (SRB) and inhibits biofilm formation, which is both corrosion agents in the reservoir [56,57]. For instance, bacterial species, such as *Bacillus licheniformis* and *Pseudomonas aeruginosa*, have been shown to have a potential antimicrobial effect on different strains of SRB [58]. A pictorial illustration of how biosurfactants aid MEOR is shown in Figure 1

An extensive literature review on MEOR showed that anionic glycolipids are the preferred category of surfactants for MEOR because of their efficient surface-reducing properties, oil-spreading activity, and the formation of a stable emulsion with crude oil [59,60,61]. This class of biosurfactant, particularly surfactins and rhamnolipids, are produced well by *Bacillus subtillis* and *Pseudomonas aeruginosa*, respectively. These microorganisms are very common in the environment, and are very easy to cultivate in an artificial setting. Crude biosurfactants produced by these organisms can be effectively used for MEOR processes since they can influence the interfacial tension reduction between the heavy oil found in reservoirs and the displacing fluid [44]. Introducing crude biosurfactants alongside surfactant-producing microorganisms will improve performance efficiency. This is because biosurfactants are tolerant to a wide range of physicochemical and environmental changes, such as high salinity (≤20%), pH (2–12), and temperature (30–100 °C) [62,63], as evident in reservoirs. By virtue of fitness and adaptability, co-introduced microorganisms will continue to produce biosurfactants in the formation. Alternatively, indigenous microorganism flooding (IMF) serves the same purpose, and may be superior. IMF entails stimulation of microbes indigenous to the reservoir with air and balanced growth media [51,64] to produce biosurfactants and CO_2_. The produced CO_2_ will not only increase the pressure in the rock formation but will reduce the viscosity of the heavy oil and react with carbonate to increase the permeability of the formation. The effect of the elicited CO_2_ will invariably lead to more oil extraction. The choice of *Bacillus* and *Pseudomonas* in MEOR is appropriate because they thrive in extreme habitats, including reservoir rock formations characterized by high salinity, pressure, and temperature.

### 2.2. Biosurfactants for the Formulation of Fuels

Diesel fuel is popularly used in electric energy production, transportation, and factories around the globe, with the associated exhaust made up of black carbon, particulate matter (PM), nitrogen oxides (NOx), sulphur oxides (SOx), carbon dioxide (CO_2_), and carbon monoxide (CO) [65,66]. Hardware technology has been employed to reduce these pollutants, especially PM, with prohibitive cost [35]. The alternative solution lies in fuel-based technology that will reduce these pollutants without compromising performance nor compensating for the engine’s performance. In pursuant to this, several research works have been carried out on the use of diesel–water blends. The diesel–water blend is the same as a water-in-diesel emulsion (WIDE), which both have the potential to reduce nitric oxides (NO_x_) and particulate matter (PM) emission while simultaneously improving the performance level. However, phase separation tends to occur in WIDE after a long period of time in storage conditions [38]. Consequently, surfactants are needed to stabilize the emulsion, with a view to ensuring that the dispersed water droplets remain in suspension within the diesel. In such a stabilized form, different additives can be added to the blend to improve performance. Currently, fatty acid esters, alcohol ethoxylates, sorbitan monooleate, tween 20, tween 80, span 80, Gemini, fatty acid ethoxylates, etc. are popular surfactants used in stabilizing diesel emulsion [67,68]. Those surfactants exhibit HLB values that range from 9 to 10 and their amount in the blend is between 0.5% and 5% by volume [35] while the water content is between 5% and 15% *w*/*w* [69].

However, synthetic surfactants are expensive and serve as environmental pollutants of concern, hence the need for a cost-friendly and sustainable alternative. This superior alternative chemical is a biosurfactant. An attempt was made by Leng et al. [70] to use rhamnolipids to obtain a finer glycerol/water-in-diesel microemulsion. These microemulsion fuels were stored at 4 °C without phase separation for over six months and it could be directly introduced into fuel to improve the cold flow property. Pekdemir et al. [71] earlier reported that rhamnolipid is an excellent emulsifier of diesel in both distilled water and seawater. Such a microemulsion defined by the surfactant produced by *Pseudomonas aeruginosa* AP02–1 can remain stable for up to 4 to 6 months [72]. Though the physicochemical properties of glycerol/water-in-diesel microemulsions are similar to those of diesel, the microemulsion can be formed spontaneously with a low consumption of energy. Such a fuel blend can form a super stable emulsion to contain a variety of additives, such as anti-foaming agents, anti-rust agents, ignition improvers, lubricity enhancers, and metal deactivators [73]. It is important to note that the diesel–water blend improves combustion efficiency, reduces unburned hydrocarbons, reduces particulate matter and pollutant emission, and provides the benefit of reducing costs [38]. 

From the foregoing discussion, it is instructive to say that when biosurfactants are applied well in the diesel–water blend, the best results of a synthetic surfactant (or a combination of the same) can be obtained. This is because biosurfactants, like rhamnolipid, can have a very low critical micelle concentration (CMC) [72], which is unique for water-in-oil emulsions. A species of the longer-chain heteropolysaccharides and proteins of emulsifying-type biosurfactants may be a better option [72]. While the biosurfactant plays the most influential role in emulsion stabilization, the water component via microexplosion, which plays a major role in improving combustion performance, reduces NOx emission (due to the cooling effect arising from water vaporization), reducing the formation of PM, soot, and hydrocarbons (due to the reduced rate of reaction) [35,74]. A comparative study conducted by Raheman and Kumari [75] on the biodiesel JB10 blend with water showed that that the JB10 blend is superior to the parent biodiesel.

### 2.3. Biosurfactants in Biodesulphurization

Certain heavy crude oil contains sulphur and nitrogen, which compromise the grading of hydrocarbon fuel and emits toxic gases, such as SOx and NOx, to the atmosphere. These gases have been implicated in the causation of health threats, including respiratory and cardiopulmonary disease [76]. Besides, these gases are also responsible for the cause of acid rain, which in turn facilitates the wear and tear of materials and skin cancer. The conventional method used in removing sulphur is hydrodesulphurization (HDS), which requires a metallic catalyst, high pressure, and high temperature. This method is targeted mainly for thiophene-based aromatic heterocyclanes: Thiophenes, dibenzothiophenes, and benzothiophene [77]. However, the listed thiophenes have a low desulphurization efficiency [78]. Thus, an alternative approach is used in the petroleum industry to get rid of sulphur from sulphur-laden oil. It is called biodesulphurization (BDS). It entails the use of competent microorganisms to selectively remove sulphur from organosulphur hydrocarbons to complement HDS without degrading its carbon skeleton. Some of these organisms are *Rhodococcus*, *Lysinibacillus*, *Pseudomonas*, *Sphingomonas*, *Bacillus*, *Gordonia, Acinetobacter*, *Arthrobacter, Mycobacterium*, *Klebsiella, Caldariomyces Paenibacillus*, and *Enterobacter* [79,80,81]. The availability of these sulphur-aromatic compounds to the microorganisms is one of the most challenging factors in the biodesulphurization process. The mobilization and solubilization effect can address this limitation to some extent by using surfactants. However, between the two categories of surfactants, the biosurfactants tend to be more advantageous due to its cost-effectiveness, eco-friendliness, specificity, biocompatibility, and ease of production.

**Figure 2:**
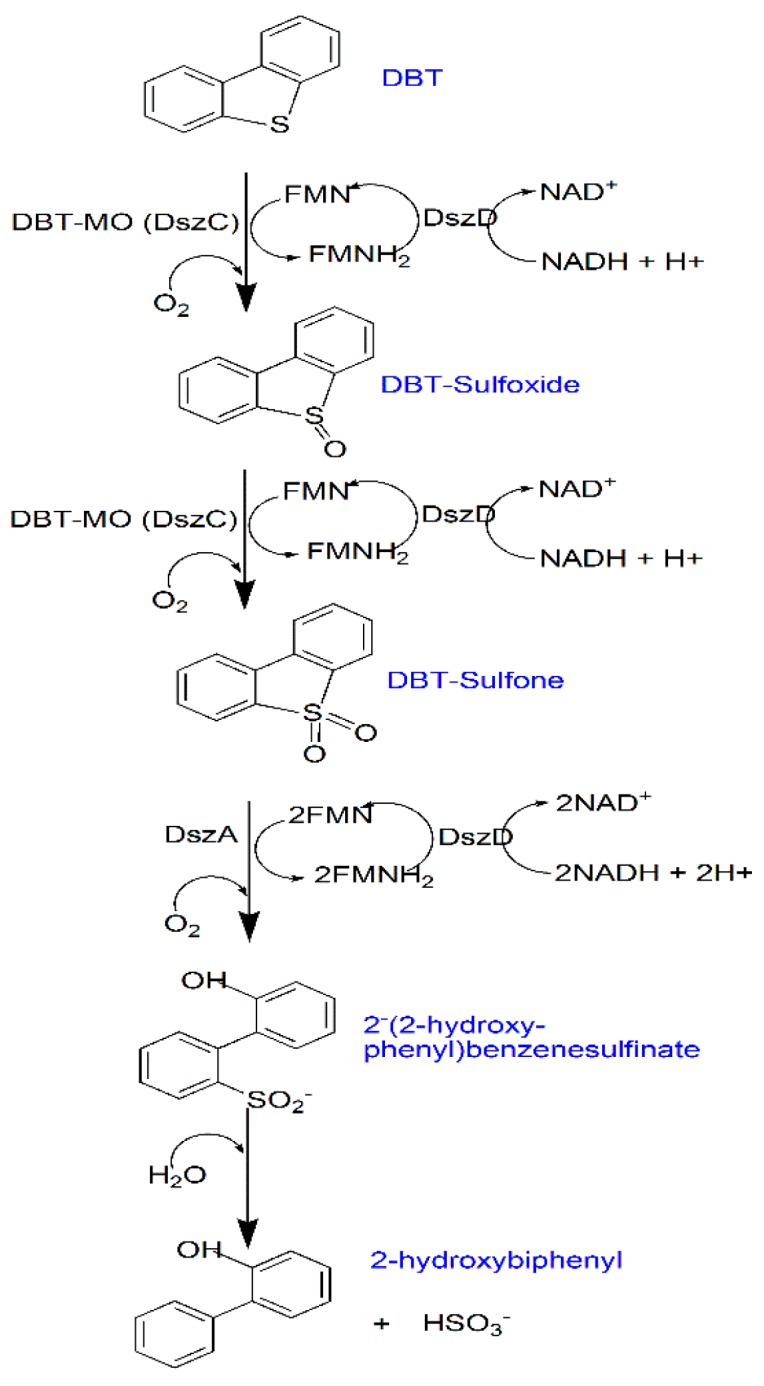
The four-step biodesulfurization (4S) pathway.

Dibenzothiophene (DBT) represents the model compound of sulphur-containing species of crude oil and fuel [82]. *Rhodococcus erythropolis* IGTS8 (a model bacterium) catalyzes DBT via what is known as the four-step desulphurization (4S pathway) in defining the BDS process (Figure 2). Briefly, this pathway involves four enzymes, including DszA, DszC (flavin-dependent monooxygenase), DszB (desulphinase), and DszD (flavin reductase). DszC converts DBT into dibenzothiophene sulfoxide (DBTO) and dibenzothiophene sulfone (DBTO_2_). DBTO_2_ is converted to 2-hydroxydioxybiphenyls-2-sulfinic acid (HBPS) by the DszA enzyme [83]. Finally, HBPS is hydrolyzed into 2-hydroxybiphenyl (HBP) and sulphite by the DszB enzyme [84]. Increased BDS activity can be enhanced by recombinant strains through the manipulation of the *Dsz* gene [85]. For example, Raheb and Hajipour [86] used an engineered biodesulphurisation biocatalyst (*Pseudomonas aeruginosa* ATCC 9027) for the BDS reaction. Using the engineered microorganism produced rhamnolipid, which lowered the energy consumption (replacing the bulk energy to the interface) in the DBT transformation process. Besides, the produced biosurfactants can resolve the mass transfer limitation of DBT through mobilization and solubilization techniques. Solubilized DBT is facilitated to approach the interface of the bacterium and be catalyzed by the enzymes from the bacterium. In a separate study, Amin et al. [87] conducted a two-stage cell bioreactor for surfactin production using *Bacillus subtilis* and the BDS process using *Rhodococcus erythropolis*. The effluent from the *Bacillus* bioreactor was fed into the *Rhodococcus* bioreactor containing DBT in hexadecane with the result being an increased BDS rate and productivity. A lipopeptide was used by Lyu et al. [88] to show that a biosurfactant can significantly increase the yield of 2-HBP. The formation of the 2-HBP marks the end of the BDS process.

By principle, one would expect the 2-HBP to partition into the hydrocarbon phase, while the S_0_ is collected in the aqueous phase alongside with the biocatalyst. This biphasic partitioning could be enhanced by the use of a de-emulsifier for purer fuel. Genetic manipulation of the genes involved in BDS obviously would increase desulphurizing activities. However, any genetic engineering approach that would regenerate cofactors means facilitating the first two steps of the 4S pathway since DszC and DszA (enzymes for the first two steps) is greatly influenced by cofactors dependent on monooxygenase [89]. It was also confirmed by Li et al. [90] that rearranging the *dszABC* operon to *dszBCA* in participating cells would give a 12-fold higher activity.

### 2.4. Transport of Crude Oil by Pipelines

Crude oil is usually transported over extended distances from the extraction fields to strategic points, such as refineries. Transportation of highly viscous crude oil has the problem of poor flowability because of the associated high asphaltenes and paraffin depositions, asphaltene muds, and plugging problems in the pipeline [91,92]. Physically, higher-capacity pumps and pipeline dimensions can be remade to restart the pipeline with higher pressure [93], heating, or the use of toluene and xylene as a choice solvent for dissolving mud. Obviously, these solutions incur the high cost of production and release highly toxic wastes into the environment [39]. In a field trial, a bioemulsifier rather than a biosurfactant was used to transport Bobscan heavy crude oil with a 200,000 cp viscosity value. The bioemulsifier was used at a concentration of two parts per thousand, relative to the oil, to form a 70% w/v oil-in-water stable emulsion. Such an emulsion can be transported for 26,000 miles [38] because it has a great capacity to stabilize the oil-in-water emulsion. A study from Amani and Kariminezhad [94] investigated an emulsion produced by *Acinetobacter calcaoaceticus* PTCC1318, which showed a positive result in cleaning the steel tubing at room temperature, indicating that it can be used in pipeline transportation.

Unlike a water-in-oil emulsion, the stabilization of an emulsion in oil-in-water in crude oil transportation via pipelines requires phase separation at its destination (refinery). Thus, interfacial tension reduction is not a priority and the tension active molecule property must have a high HLB. Thus, biosurfactants with high HLB (bioemulsifiers) are highly suitable for increasing the mobility of oil. Among bioemulsifiers, emulsan has been given the highest preference because it has a good number of reactive groups that make the molecule hold tightly to droplets of oil [95], thereby forming barriers that stop drop coalescence. Mazaheri-Assadi and Tabatabaee [39] reported the use of emulsan (amongst other powerful bioemulsifiers: Alasan and biodispersan) produced by *Acinetobacter* strains as the most effective in reducing the viscosity of oil in transit. On reaching its destination, the hydrocarbons can be treated with emulsane enzymes to remove the bioemulsifiers from the emulsion [74] or de-emulsifier to de-water the emulsion. The application of emulsan or other analogues of emulsifiers would have a challenge when a huge volume of oil is required either in producing the required quantity of the pure emulsifiers or the mechanism of mixing with the high volume of the oil. Again, where blockages have set in, the application of bioemulsifiers may have no positive effect. Hence, the application of emulsifiers may serve as a prophylactic measure to avoid deposition of a new pipeline or a physically cured one.

### 2.5. Oil Storage Tank Cleaning 

The periodical cleaning of waste and heavy oil fraction tanks presents a challenge due to deposits formed in the tank. At times, these washings are informed by a planned repair of leaking tanks. The washing of these deposits requires different conventional methods, which are hazardous, time-consuming, laborious, and an equally expensive procedure [96]. Currently, the washing operation may involve solvent liquification, hot water spraying, and land farming disposal [97]. With microbial biosurfactants, the oil-in-water emulsion forms, thereby decreasing the viscidity of sludge and oil deposits to facilitate the pumping of waste. A study by Saeki et al. [98] reported how sludge from a the bottom of a tank was treated by using biosurfactant JE1058BS, (from *Gordonia* spp.), with a superlative result that remained effective for 21 days. Later, another study from Diab and Din [99] investigated the effect of the supernatant from *P. aeruginosa* sp. SH 29 applied to the cleaning of oil-contaminated vessels, and discovered that in 15 min, oils was recovered from the bottom and walls of the vessels, and floated on the supernatant as a distinct phase. Further, Silva et al. [100] confirmed that *Pseudomonas cepacia* CCT6659 biosurfactant could be used for cleaned beaker walls contaminated with an oil layer of 80%, which gives credence to this biosurfactant as an oil storage tank cleaner. The application of biosurfactant in the tank bottom sludge forms an oil-in-water emulsion and consequently reduces the viscosity of the oil deposits. At the reduced viscosity, the pumping of the oil deposit is thus greatly facilitated [95]. Apart from this cleaning purpose, crude oil can also be recovered; however, only when the emulsion is broken. This can be facilitated by the use of de-emulsifiers.

## 3. Biosurfactants for Environmental Remediation

The petroleum industry releases waste generated from its three chains: Exploration/production, refining, and transportation. These wastes include, among other things, drill waste, produce water, oil spills, tank bottom sludge, effluents, gas emission, and oil sludge from maintenance operations. These wastes are received by different eco-settings, including atmospheric air, terrestrial water, and land systems. Common pollutant components received by these different environmental media are polyaromatic hydrocarbons (PAHs), waxes, asphaltenes, monoaromatic hydrocarbons (BTEX), paraffin, and heavy metals. These pollutant species present themselves as a global concern due to their toxigenic effect on healthy microorganisms, plants, animals, and humans. Consequently, the need to remediate them becomes pertinent in different environmental media. Different methods of treatment are available, ranging from physicochemical to biological [101]. The biological method, denominated as bioremediation, is trendy because of its eco-friendliness, cost-effectiveness, and simplicity. Across all variants of bioremediation, bioavailability remains the first point of consideration. This is where surfactants come into play in environmental remediation. This class of chemicals represents itself as either synthetic or natural. Both classes of surfactants are compatible with different waste-inundated environments: Soil and aquatic systems.

### 3.1. Bioremediation of Hydrocarbon-Contaminated Marine Environments

The inadvertent spill of hydrocarbons during transport and leaks from drilling rigs into aquatic bodies, such as lakes, ponds, bays, and oceans, is enormous [102]. The consequences, too, are enormous: Fundamental disruption of the aquatic food chain, death of aquatic lives, poor penetration of sunlight, etc. Remediation of the polluted marine water body is a necessary response that needs to be undertaken urgently. Conventional methods require the application of dispersants (with complex additives, including surfactants) to form fine droplets out of oil slicks and oil-in-water emulsion from mousse oil [103]. However, these chemical dispersants are toxic to various aquatic lives and are hardly biodegradable [104]. Thus, an eco-friendly, biodegradable, and effective dispersant is required. Biosurfactants could serve these purposes in addition to their cost-effectiveness. The marine ecosystem provides potential habitats and niches for diverse microorganisms [105], including hydrocarbonclastic. In addition to an introduced biosurfactant, more surface-active compounds are elicited in the course of utilizing the exposed hydrocarbon droplets as a source of carbon and energy [106]. Marine bacteria that have been cited as biosurfactant producers, as well as being hydrocarbonoclastic in nature, include *Alcanivorax*, *Halomonas*, *Rhodococcus*, and *Pseudomonas Bacillus*, amongst others [107,108].

An investigation conducted by Whang et al. [109] using rhamnolipid and surfactin revealed that a 40-mgL^−1^ addition of surfactin in the medium enhanced biomass growth, with 90% diesel degradation compared with 40% degradation in a control batch experiment. A decrease in both biomass and degradation ensued when the concentration of biosurfactants was above 40 mgL^−1^. The addition of rhamnolipid to diesel–water systems from 0 to 80 mgL^−1^ increased biomass growth and diesel degradation. Besides, biosurfactants produced from *Ralstonia picketti* and *Alcaligenes piechaudii* have also proved effective in the degradation of hydrocarbons by up to 80% [110]. Another study by Feng et al. [104] recorded a dispersant effectiveness of lipopeptide produced by *Bacillus subtilis* HSO1121 at a low surfactant:oil ratio. Besides, the biosurfactant excellently stimulated microbial oil degradation. A study from Shah et al. [111] formulated a binary mixture of sophorolipid and choline laureate as an effective dispersant, which was better than the individual surfactant, for oil spill remediation.

The biosurfactant dispersant not only increases the surface area (formation of micelles due to emulsification) but also increases the solubility and mobility of hydrocarbon pollutants. On the aspect of microorganisms (especially bacteria), the biosurfactant induces cell surface alteration to be more hydrophobic, thereby raising the pinocytosis index of hydrocarbons by the microorganisms [102]. It is instructive to note that the toxigenic effect of the hydrocarbons selectively shifts the microbial community to favor autochthonous hydrocarbon-degrading organisms [112] in the impacted environment. Thus, the adapted microorganisms utilize soluble and bioavailable hydrocarbons for cell growth and proliferation. In the process, the quantity of the spilt hydrocarbons becomes drastically attenuated in the presence of a contributing consortium of bacteria and fungi. However, when the optimum concentration of the biosurfactant is exceeded, biomass growth and the degradation rate will be negatively affected. A critical examination of the biosurfactant dispersant used in marine remediation indicates that a biosurfactant with low CMC is effective while a concentration of the dispersant beyond 1 to 1.5 CMC of the biosurfactants becomes ineffective for biodegradation enhancement [9,102,109]. Beyond the optimum concentration, biosurfactants cover biosurfactant–hydrocarbon aggregates, thereby preventing microorganisms from accessing hydrocarbons for utilization [113] or the presentation of biosurfactants as a preferable substrate [114]. 

### 3.2. Bioremediation of Hydrocarbon-Contaminated Soil 

Soil serves as a repository to hydrocarbons from oil and gas industry-related activities, such as exploration and production, leakages from underground/aboveground storage tanks, pipeline leakages, effluents, and industry- and transportation-related accidents [115]. The hydrocarbon pollutants constitute alkane, cycloalkane, monoaromatic hydrocarbons (MAHs), polyaromatic hydrocarbons (PAHs), resins, asphaltenes, and heavy metals. Apart from toxigenic effects, they also possess physicochemical properties that make them insoluble and recalcitrant. Assorted means of remediation in soil exist, which include physical, mechanical, chemical, and biological methods [116]. Amongst them, the biological method stands out because it drives the remediation process on a natural course, using renewable organic resources and low technology. These renewable organic resources include plants, microorganisms, and surfactants. Surfactants from these organisms, especially from microorganisms, have a critical role to play in the remediation of soil contaminated with hydrocarbons.

**Figure 3:**
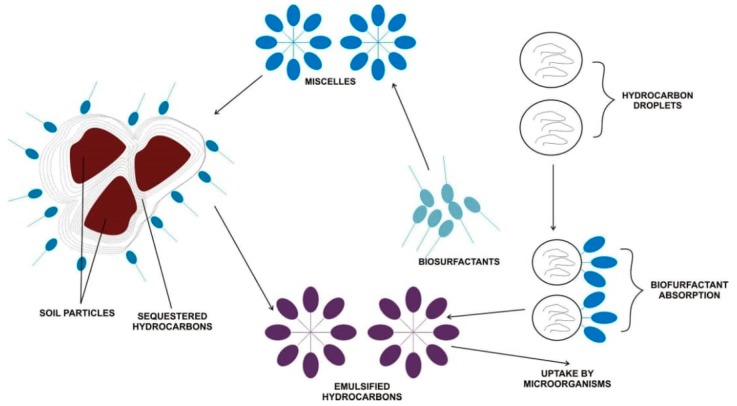
Hydrocarbon and biosurfactants interaction with soil during the bioremediation process Adapted from [117].

Bioremediation of a hydrocarbon-contaminated environment, like soil, depends on the bioavailability of the hydrophobic compounds. This could be achieved through different mechanisms, which include modification of microbes’ cell surface, solubilization, and the desorption of pollutants [117]. A study conducted by Shin et al. [118] used a rhamnolipid to remediate phenanthrene-contaminated soil by the combined solubilization–biodegradation regime. In the solubilization step, an appreciable percentage of the contaminant was removed and a significant decrease of phenanthrene was recorded during the degradation stage. Further, Bustamante et al. [119] noted the influence of alasan on the biodegradation of polyaromatic hydrocarbons (PAHs). The rate of fluoranthene mineralization was above 50% (using 500 μg ml^−1^ of alasan), with an attendant significant increase in the rate of phenanthrene mineralization by *Sphingomonas paucimobilis* EPA505. Similarly, Jorfi et al. [120] recorded 86.4% of pyrene degradation with an initial concentration of 500 mg/kg. Complete degradation of aromatic hydrocarbon was demonstrated using a chemical surfactant (FinasolOSR-5) combined with a trehalose lipid biosurfactant [121]. A simplified mechanism of biosurfactant action is illustrated in Figure 3 for a better understanding of hydrocarbon bioremediation in soil. 

Hydrocarbons usually have a high octanol-water coefficient constant ratio, which increases with molecular weight. This value resonates with their insolubility and insensitivity to degradation. The presence of a biosurfactant and its property of mobilization, emulsification, and solubilization makes the hydrophobic organic matters, including hydrocarbons, be soluble and bioavailable. In the presence of biosurfactants, the contact angle of the soil–oil system increases but reduces the capillary force binding the soil and oil together [122]. This defines the mobilization process and occurs below the biosurfactant’s critical micelle concentration (CMC). The solubilization mechanism ensues above the CMC with the formation of micelles and increases the solubility of hydrocarbon [123]. The soluble hydrocarbon can be made available to cells (bioavailability) by emulsification of the non-aqueous phase liquid contaminants and facilitated transport of the pollutants in the solid phase. Bioavailability greatlys enhance microbial degradation and phytoremediation of hydrocarbons in the soil matrix, given that all other environmental factors and nutrients are optimal. Also, the absorption of biosurfactants to hydrocarbon particles decreases the path length of diffusion between the contaminant and microorganisms [9], increases the uptake of hydrocarbons by microorganisms, and enhances enzyme activity in the soil [113]. Though subject to more robust research, it has been reported that rhamnolipids produced by the *Pseudomonas aeruginosa* strain specifically degrade hexadecane, indicating that a specific biosurfactant does degrade a particular type of hydrocarbon [108]. Rhamnolipid has been reported in the remediation of diverse kinds of hydrocarbon more than any other biosurfactant [120].

### 3.3. Soil Washing

Soil washing is an ex-situ remediation technique that separates hazardous compounds from (excavated) soil by washing of the contaminated soil with a liquid often incorporated with chemicals. The main aim of soil washing is to remove contaminants that bind to fine-grained soils like clay, silt, sand, and gravels [124]. The wastewater can then be treated and finally disposed of while the washed soil can be reused as backfill at the excavated site. The technique can be applied to soil contaminated with fuels, metals, semi-volatile organic compounds, and pesticides. Washing fluid may be composed of water, water/chelating agents, water/surfactants, bases or acids, or organic solvents [125] depending on the target contaminant. Waste of organic compound origins and chlorinated hydrocarbon contaminants are best removed by soil washing. The reason for the surfactant is to increase the solubility of non-aqueous phase liquids (NAPLs) through the reduction of the surface tension between the contaminant and the soil particles [126]. Common synthetic surfactants used for soil washing are Tween 80 (a non-anionic model), sodium dodecyl sulphate (an anionic model), and alkylbenzyldimethylammonium chloride (a cationic model). However, in recent times, biosurfactants have been used in soil washing technology mainly because of their ‘green’ advantage. It is important to note that if the biosurfactants are labile, it is not an efficient option in soil washing [127]. 

Rhamnolipids have been confirmed as soil-washing agents for improved removal of hydrocarbons and metals. Rhamnolipid-enhanced soil washing targeted for hydrocarbon results from mobilization and solubilization [30,128] to facilitate separation of the pollutants from the solid particles and increase the partition of the contaminants in the aqueous phase [129]. A study from Ochoa-Loza et al. [130] proved that monorhamnolipid sorption on soil matrix constituents is concentration dependent and that the monorhamnolipid formed sorbs more strongly alone compared to mixed rhamnolipids. Lai et al. [131] did a comparative study and proved that rhamnolipid removed total petroleum hydrocarbons from heavily polluted soil up to 63% against surfactin (62%), Triton-100 (40%), and Tween 80 (35%). Similarly, Conte et al. [132] carried out a comparative study between humic acid and synthetic surfactants (SDS and TX100) alongside water in the washing of polluted soil. They were able to prove that the organic surfactants removed pollutants up to 90%. Hence, the application of natural humic acid solutions seems to be a better choice for the washing of highly polluted soils because of their additional microbial activity promotion capacity, unlike chemical surfactants. Though some level of success has been recorded with biosurfactant application in soil washing, soil sorption remains a key limitation to the application of biosurfactants.

Soil organic matter (SOM) is the most influential factor that governs hydrocarbon sorption to soil particles, though pH, soil texture, clay minerals (smectite, illites, and kaolinite being the most common), and cation exchange capacity also contribute [133]. The presence of biosurfactants can desorb hydrocarbons but is higher in freshly polluted soil than aged soil due to their solubilization property at a concentration higher than their CMC in the oil-in-water system, which results in an increase in the mass transfer of the pollutants from the oil phase to the aqueous phase. Prior to the solubilization stage, the biosurfactants, by virtue of their surface/interfacial tension reduction, address the mobilization phase with markers, such as capillary force (the force that holds the oil and soil) reduction, wettability, and contact angle reduction [134]. This happens at a concentration below the CMC of the biosurfactants. The mobilization mechanism of the biosurfactant depends on the ionic charge of the biosurfactants [134,135,136,137]. Thus, the concentration of the biosurfactant may be reduced in the process. An anionic biosurfactant would perform better as a washing agent [138] than a cationic or nonionic surfactant and the produced anionic wastewater is easier to destabilize through the charge neutralization mechanism. A desorbing medium other than a biosurfactant (which has a similar structure and composition) can be used alongside with the biosurfactant to enhance the desorption of hydrocarbons from polluted soil for better result [133]. In the case of loss concentration, a calculated homogenous biosurfactant can be periodically introduced in the washing system to achieve the remediation goal [122]. Rhamnolipids are among biosurfactants that have met most of these requirements for use as a washing agent in the soil washing technique. This is supported by the good number of published articles on the use of rhamnolipid for soil washing [139,140].

### 3.4. Metal Bioremediation

Metals are persistent soil contaminants and constitute varying degrees of health hazards to animals and humans. Metal contamination has been linked to mental and physical retardation, birth defects, cancer, liver and kidney damage, learning disabilities, etc. [141]. Remediation of soil contaminated with toxic metals, such as lead, cadmium, zinc, and chromium, has been achieved by landfilling [142]. Currently, renewed interest in utilizing microorganisms to effect in-situ remediation of metal-contaminated surface and subsurface soils has been intensified due to the high cost of conventional remediation [9]. The goal of surfactant utilization for both organics and metals is similar: To increase the solubility of the contaminant of interest to facilitate the removal by degradation or flushing. However, it is instructive to note that there are some key differences between metal-contaminated and organic-contaminated soils that need to be considered. Unlike organic contaminants, heavy metals are not biodegradable and are mostly found as a cationic species [143]. Metal pollutants can either be removed or immobilized (being transformed from one chemical state to another, either by a redox process or alkylation, as a result changing in their mobility and toxicity potency) [144,145]. 

Like other pollutant remediation, the use of eco-friendly approaches in metal remediation is currently being pursued, which demands the use of renewable resources like plants, microorganisms, and biosurfactants for obvious reasons.

Biosurfactant-induced remediation of metals adopts different mechanisms, including sorption, desorption, and complexation [146]. Microbial surfactants have been employed both in soil washing and pump-and-treat techniques to assist in the dispersal, desorption, and solubilization of metals in polluted soil and groundwater. Research carried out by Ochoa-Loza et al. [147] reported rhamnolipid-metal stability constants to be similar or higher than stability constants recorded by Pb^2+^, Zn^2+^, Fe^3+^, Ni^2+^, and Mn^2+^ with organic acids used for conventional metal complexation. Franzetti et al. [148] noted that desorption of metal by a biosurfactant depends on the complexation formation in line with Le Chatelier’s principle and mobilization based on interfacial tension reduction. Overall, the mechanisms driving biosurfactant–metal binding are precipitation–dissolution, counter-ion association, electrostatic interaction, and ion exchange [149]. More information about soil washing for metal removal can be found in the review done by Delil and Koleli [150] and Wuana and Okiemen [142]. Kim and Song [151] used the soil washing method using a flocculating agent to remediate 88% of Cs. The rhamnolipid-aided washing method was proven by Nielson et al. [152] to be more efficient than a synthetic surfactant (carboxymethyl-β-cyclodextrin). A sophorolipid-enhanced soil washing method was used to remove 83.6% of Cd and 44.5% of Pb by Qi et al. [153]. However, in the presence of sophorolipid-producing *Starmerella bombicolla*, the removal increased to 95% of Cd and 52% of Pb. Liduino et al. [154] used biosurfactant-aided phytoremediation to efficiently remove Ni (41%), Cr (30%), Pb (29%), and Zn (20%). Sarubbo et al. [155] used crude biosurfactant extracts from *Candida guilliemondii* UCP 0992 to remove 98.8% of ZN, 89.3% of Fe, and 89.1% of Pb. These pieces of evidence demonstrate that natural tensioactive biomolecules can play a significant role in metal removal in a contaminated ecosystem, especially soil.

**Figure 4:**
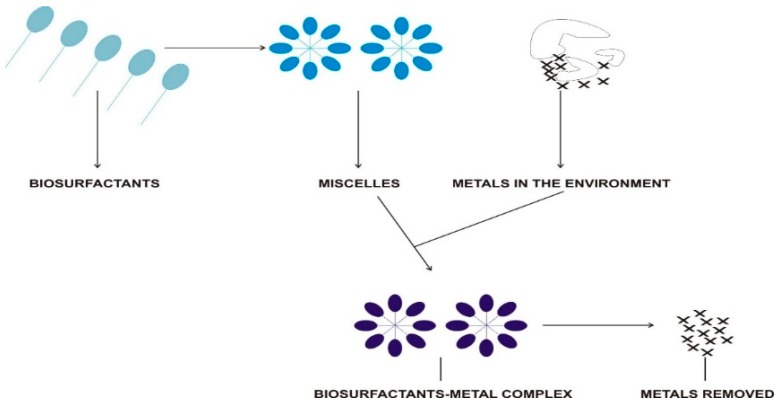
The removal of metals through the mediation of biosurfactants.

Since contaminant sorption relies on the chemical properties of both the soil and the metal, the choice of surfactant used for contaminant complexation is essential [143]. The addition of a biosurfactant could promote desorption of heavy metals from its solid phases. As a principle, an anionic biosurfactant forms electrovalent bonds with the metals, thereby resulting in nonionic complexes stronger than that between soil and metal. The complexes thus formed with the biosurfactant desorb from the soil matrix and migrate to the soil solution and subsequent incorporation into micelles (Figure 4). Though in the absence of micelles, metal can still be desorbed because the rate-controlling mechanism is the surface reaction step. The mechanisms would either form outer-sphere or inner-sphere complexes that could be facilitated by oxide protonation/deprotonation in the presence of water molecules. Within this working framework, the presence of foreign cations and a high salinity will drastically reduce the efficiency of the complexation mechanism [156]. Rhamnolipids and surfactins have been shown to be popular biosurfactants used for metal remediation. The addition of adapted microorganisms would have a positive effect on the overall success of the metal remediation process. Microorganisms can indirectly influence the mobility of metal by adjusting the pH or by stimulating substances, which could change the mobility of the metals [122]. Studies have shown that nickel toxicity is reduced by increasing the pH by a variety of different organisms, including yeast (*Cryptococcus terreus*), filamentous fungi (*Penicillium vermiculatum*, *Rhizopus Stolonifer*), and bacteria (*Serratia marcescens*) [122]. Explanations for this detoxification process include the high pH conditions, and microorganisms also have the capacity to take up or adsorb a great amount of the metal ions through metabolism-dependent uptake [157].

## 4. Application of Biosurfactants in Agriculture

Improved soil quality is a prerequisite for agricultural activities and crop production. Soil quality for agricultural use is affected by the presence of inorganic and organic pollutants, which affect the biotic and abiotic components of the soil [158]. To improve the quality of such impacted soil, remediation is needed to reduce organic and metal pollutants to an acceptable or tolerable level as seen in the previous section. Plant growth in healthy land needs interaction with soil microorganisms in the rhizosphere. This plant–microbe interaction is essential for both plants and microbes, especially bacteria [159]. Factors that aid these interactions are biofilm formation on the root surface, the release of quorum-sensing molecules, and microorganism motility [160]. This symbiotic relationship influences nutrient availability and uptake that is critical for plant growth promotion [31]. According to Ma et al. [161], plant growth-promoting microorganisms (PGPMS) alleviate metal phytotoxicity, stimulate growth through the induction of defense mechanisms against pathogens, and change metal bioavailability in soil via acidification, chelation, precipitation, complexation, and reduction–oxidation reactions. Interactions of pests and pathogens overwhelm plants in natural ecological settings. Human intervention in pest/pathogen control, enhancement of plant–microbe interaction, and soil remediation is key, with a view to maximizing crop yield and turnover. Conventionally, synthetic chemicals are used in all these areas, with environmental degradation and health risks having a heavy toll. To lessen the burden of agro-chemical pollutions and health issues arising from them, the quest for green technology becomes imperative. A green molecule that has such a multifunctional application to address the raised concern is a biosurfactant.

Biosurfactants have been shown to play a huge role in the bioremediation of hydrocarbons, metal detoxification and/or removal, and soil washing technology [112,113,117,119,120,135,147,148,150]. Research conducted by Sachdev et al. [158] reported that biosurfactants aid nutrient uptake, including root cell differentiation. Further, biosurfactants produced by root-associated bacteria increase nutrient availability and uptake and support the efficient distribution of metals and micronutrients in the soil, thus aiding plant growth promotion [31], protecting against toxic substances, and serving as a carbon source. Several biosurfactants have biocontrol value for sustainable agriculture because these molecules have antimicrobial activity against plant pathogens [158]. Biosurfactants produced by *Pseudomonas putida* were proven to lyse zoospores of *Phytophthora capsici*: The causal agent of the damping-off of cucumber [162]. Biosurfactants produced by strains of *Pseudomonas fluorescens* were proven effective against *Pythium ultimum*, *Fusarium oxysporum*, and *Phytophthora cryptogea*, which are notorious plant pathogens [152,163]. Biosurfactants also inhibit aflatoxin production by *Aspergillus* spp., which infects crops, such as peanuts, cottonseed, and corn, during storage [158]. Thus, biosurfactants play various roles in plant pathogen elimination in agriculture and in different processes. Conventional arthropod control strategy involves applications of broad-spectrum chemicals and pesticides, which often produce undesirable consequences. Therefore, innovative approaches need to be sought to address the high cost of chemical control and the chemical resistance of insect populations associated with the conventional system. Lipopeptide extracted from several bacteria are active against fruit fly *Drosophila melanogaster*, and hence can be used as a biopesticide [164]. Di-rhamnolipids, according to Kim et al. [165], possess insecticidal potential against the green peach aphid. In addition, Parthipan et al. [166] reported how biosurfactants from *Bacillus subtilis* A1 *Pseudomonas stutzeri* NA3 inhibited young instars of *Anopheles stephensi* and reduced the longevity/fecundity of adult mosquitoes.

**Figure 5:**
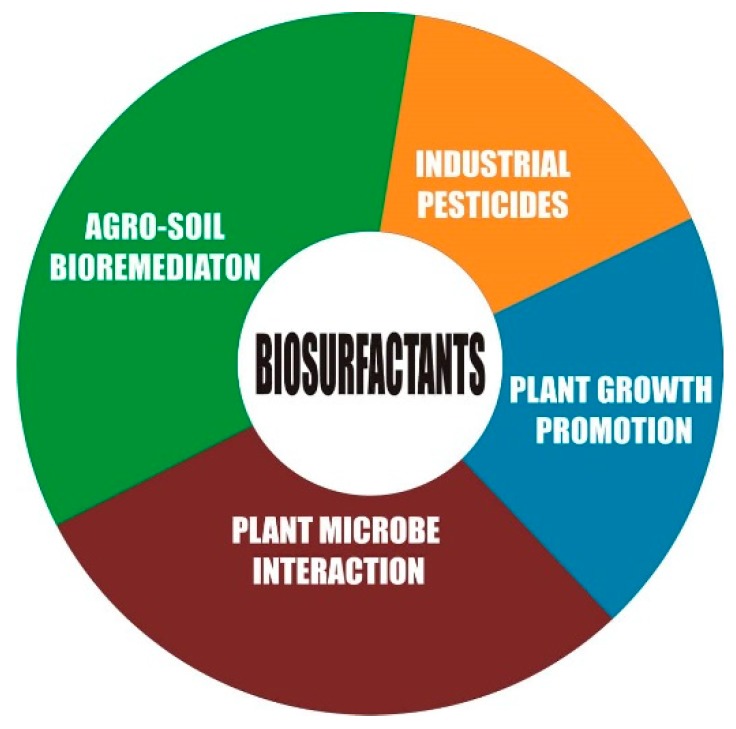
Application of biosurfactants in the agriculture sector.

Biosurfactant properties, such as low CMC, interfacial surface reduction, and emulsification, which influences mobilization and solubilization, play a vital role in metal and hydrocarbon remediation. More so, the wettability property of biosurfactant counters the micronutrient-poor solubility created by soil organic matter content, adsorption surface, pH, nutrient interaction, and soil texture [31], thereby facilitating the availability and uptake of nutrients. The working principle is that biosurfactants chelate the trace metal hitherto sorbed to the soil and desorb and remove the metal from the soil, which then becomes incorporated into the micelles [149]. Once the nutrients become available for adsorption, the sustainability is assured because the biosurfactant would hardly be affected because it is tolerant to the fluctuation of environmental factors, such as salinity, pH, pressure, temperature, etc. The assembly and maintenance of the plant holobiont or phytomicrobiome are driven by biomolecular cues—the quorum-sensing chemicals, root exudates, and microbial signals [161]. These quorum-sensing molecules—acyl-homoserine lactones (AHLs)—contribute to the regulation of exopolysaccharide (EPS), essential for the formation of biofilm [167], which can play the role of metal desorption. Root exudates have the potential to enhance the bioavailability of metals and nutrients, and act as a carbon/energy source for microbes. Consequently, the microbial mass is increased in the rhizosphere with the release of biosurfactants. The biosurfactant can regulate AHLs, for example, rhamnolipids from *Pseudomonas aeruginosa* [168], as well as enhancing phytoremediation. In turn, these microbes stimulate exudation from the plant roots [168]. Free-living microbes are able to take advantage of plant exudates to produce diverse organic compounds different from the exudates, such as volatile organic compounds (VOCs), Myc factors, Nod factors, and exopolysaccharides [161]. VOCs, by virtue of their chemical nature, trigger plant defense and growth promotion mechanisms for the colonization of nutrient-deficient soils. Biosurfactants’ ability to lyse and exhibit an inhibitory effect against certain organisms makes them antimicrobial. The biopesticide value of biosurfactants lies in the fact that the molecular signal leads to defense genes and the accumulation of antimicrobial metabolites [169]. These molecular signals are called microbe-associated molecular patterns (MAMPs). Glycolipids, especially rhamnolipids, have been given the most attention in agricultural applications [165]. Figure 5 shows the role biosurfactants play in agriculture.

## 5. Biosurfactants in Other Industries

Apart from the petroleum industry, environmental remediation, and agriculture, biosurfactants are used in other industries, such as laundry detergents, medical/pharmaceuticals, food industry, textile, paint, leather, paper, mining, nanotechnology, bioprocessing, and recently in energy-saving technology [20,25,26,38,170,171,172,173,174,175,176,177,178,179,180,181,182,183,184,185]. The multifunctionality and application versatility lies in their properties (surface and interfacial tension reducing ability, low CMC, wettability, specificity, antimicrobial activity) and their advantages (environmental friendliness, biodegradability, biocompatibility, low toxicity, ease of production, chemical diversity, and cost-effectiveness). These unique natures of the biosurfactants allow their utilization and possible replacement of chemically synthesized surfactants in various industrial operations. However, it is worth knowing that biosurfactants command only ca. 2.5% of the global surfactant market [186], even though its global demand is growing appreciably. The low output of biosurfactants is a result of the low productivity and downstream processing cost [29,33,34,115,187].

In the midst of low output, experimental evidence has proven that biosurfactants can be used in the medical/pharmaceutical sector, functioning as an antimicrobial agent [188,189], anticancer agent [21,23], anti-adhesive agent [20,24], immunological adjuvants [22,25], antiviral agent [16,190] and gene delivery agent [15,191]. Microbial biosurfactants play a functional role in the food industry as a food emulsifier and stabilizer [192], foaming agent, adhesive, and wetting and antimicrobial agent [190]. Fracchia et al. [175] conducted a detailed review of biosurfactant applications in the textile industry where they are used as a pretreatment agent, for dye solubility, and to achieve penetration of the fiber. In the leader, paint, and papermaking industry, biosurfactant has been used as a degreasing, dispersant, defoaming, deresinification, calendaring, coating, and color levelling agent [178,181,192]. Biodispersan produced from *Acinetobacter calcaoaceticus* A2 was used to disperse a 10% limestone in a water mixture and also prevented flocculation [185]. Biosurfactant at an alkaline pH lowered the energy required for cleaving the microstructure of limestone. Also, biosurfactants have been proven to have potential in metallic nanoparticle synthesis [171]. Eswari et al. [193] synthesized silver nanoparticles from AgNO_3_ using surfactin from *Bacillus subtilis*. A blend of biosurfactant and nanoparticles serves as a dual function recovery mechanism for oil recovery [194]. Similarly, Balakrishnan et al. [195] used biosurfactant to optimize the synthesis of polyethylene nanoparticles. The use of biosurfactant aided a reverse micelles system to recover antibiotics, enzymes, and proteins, which [170,180] is relatively new but shows high promise, with the potential for high-scale production and continuous operation. The application of biosurfactants in energy-saving technology with respect to eco-ice systems has been less publicized. Kitamoto et al. [196] used biosurfactant to achieve a 35% ice-packing factor (IPF) at a concentration of 10 mgL^−1^ in comparison to Span 80 (sorbitan monoocleate), a synthetic surfactant, which scored 30% IPF at a concentration of 1000 mgL^−1^. The use of biosurfactants in detergent laundry and cosmetics cannot be overemphasized.

In the commercial laundry detergent, cosmetics, and other household and personal care industry, ecological issues and the need for green solutions influence the increasing demand for biosurfactants [197]. Household and personal care products record more than 60% of biosurfactant application followed by industrial cleaners and petroleum biotechnology [198]. This could be attributed to the amenability of the commonest biosurfactants classes: Glycolipids, lipopeptides, and polymeric surfactants. 

Rhamnolipid, surfactin, and sophorolipids have shown to be effective in functions that rely on solubilization, thus they are suitable in MEOR, biodesulphurization (in the presence of the active desulphurizer), agriculture, soil washing, and water-in-oil blend. Rhamnolipids are more active in the desorption of materials in the soil. Table 1 gives a summary of the industries in which different types of biosurfactants have been applied lately. Bioemulsifiers (polymeric surfactants) show a better efficiency when it comes to transportation of heavy crude oil. MEL-A is unique for gene delivery due to its self-assembling actions. The fact that microorganisms (*Pseudomonas aeruginosa*, *Bacillus subtilis*, *Candida* spp. *Acinetobacter calcoaceticus*) producing these popular biosurfactants are not fastidious in nature gives the hope that in the future they will compete with synthetic surfactants. What needs to be done is intensified research to optimize the growth conditions of these microbes, genetically manipulate them to become hyper-producers, and find out the most cost-effective growth resource for use and recovery.

## 6. Conclusion

The surfactants of bacterial, fungal, and yeast origin are referred to as microbial biosurfactants. Although, synthetic surfactants are widely used in industrial applications because of their availability in commercial quantity, unlike microbial surfactants. The use of synthetic surfactants in various industries is highly associated with huge environmental impact and undesired ecological disturbances. These drawbacks can be resolved through the use of biosurfactants in place of their synthetic congeners in addition to their favorable competitiveness and greener value. With properties, such as eco-friendliness, specificity, low toxicity, stability in varying environmental conditions, and chemical diversity, microbial biosurfactants stand the chance of replacing synthetic surfactants in industrial applications (such as the petroleum industry, bioremediation, agriculture, medicine/pharmaceuticals, food industry, laundry, cosmetics, and energy-saving technology) in the near future. The most widely used biosurfactants are rhamnolipids (from *Pseudomonas*), sophorolipids (mainly from *Torulopsis*), mannosylerythritol lipid (mainly from *Candida*), surfactin (from *Bacillus*), and emulsan (from *Acinetobacter*). Their use in different biotechnological applications will reduce environmental pollution that is currently caused by synthetic surfactants, thereby engendering sustainability. Currently, only a few small industries are producing microbial biosurfactants for commercial use in the global sector. Large industries should take a solid step to incorporate microbial biosurfactants in their commercial products to enhance the use of biosurfactants in the global market. Although biosurfactants are thought to be ecofriendly, few research findings indicate that under certain circumstances they can be reversed to being toxic in the environment. Nevertheless, careful and controlled use of these interesting surface-active molecules will surely help in the enhanced cleanup of toxic environments and provide us with a clean environment. 

## Figures and Tables

**Table 1:** Industries where biosurfactants are applied: Medicine/pharmaceuticals, petroleum industry, agriculture, cosmetics, and laundry detergents.

Industry	Field	Biosurfactant	Mechanism/Functioning as/Property Used	Reference
Petroleum biotechnology	Extraction of crude oil from reservoirs	Glycolipids and Lipopeptide	Biosurfactants enhance the formation of stable water-oil emulsion, break down oil film in the rock and reduces tension/interfacial tensions thereby reducing the capillary forces that impede oil movement through the rock pores	[38,43]
	Transport of crude by pipelines	Emulsan, alasan, biodispersan	High molecular weight biosurfactants form a stable water-in-oil emulsion which aids oil mobility, viscosity reduction and prevents drop coalescence	[72,199]
	Oil storage tank cleaning	Rhamnolipids	A well-circulated biosurfactant will form an oil-in-water and lift/mobilize oil sludge from the bottom of the tank and solubilize in the already formed emulsion	
Bioremediation	Spill remediation (aquatic)	Glycolipid and Trehalose Lipids	Solubilization, oil bioavailable to hydrocarbon-degraders and longer shelf life, biodegradability	[200]
	Soil washing Wastewater treatment	Rhamnolipids Lipopeptides	Reduction of surface and interfacial tensions lead to mobilization and consequent removal of oil from the soil Physically separate, concentrate and remove chemicals of concern for modification, recycling or disposal. Rely on detergency, act as emulsifiers/de-emulsifiers and as a bioavailability enhancer	[134,201]
	Hydrocarbon remediation (soil)	Rhamnolipids, sophorolipids, surfactins	The solubilization property enhances the distribution of contaminants into the aqueous phase, thereby increasing the contaminant bioavailability for biodegradation	[202]
	Heavy metal remediation	Rhamnolipids	Metal-removal mechanisms by biosurfactants from soils are complexation, ion exchange, electrostatic interactions and counterion binding resulting in metal desorption, metal mobilization and metal entrapment by micelles.	[30,203]
Mining Nanotechnology	Precious metal recovery Silver and gold nanoparticles	Biodispersan EPS from algae	Lowers the energy required for cleaving the microstructure of ground limestone. Utilize solubilization property and act as a sequestering agent Biosurfactant producing organisms converts (Ag-Au) NO_3_ to silver/gold particle using enzyme such as nitrate reductase.	[176,185,193]
Agriculture	Improvement of soil quality	Glycolipid	Consideration of all the soil-related bioremediation	[204]
	Plant pathogen elimination	Rhamnolipids, cyclic lipopeptides	The biosurfactants act on the target cell by disrupting cell surface structures, thereby liberating the intracellular contents of the plant pathogen	[205]
	Plant-microbe interaction	Rhamnolipids	The establishment of the plant-microbe interaction is dependent on the exchange and sensing of a variety of signals (biosurfactants inclusive) by both types of partners.	[158,206]
	Pest control	Lipopeptides by *Bacillus subtilis*	Detergency property of biosurfactants exhibit toxicity against nematodes and insects	[189]
Medicine/Pharmaceuticals	Gene delivery Antimicrobial activity	MEL Anionic surfactin isoform, rhamnolipids	Cationic liposome bearing MEL-A effectively increased the transfection of genes into mammalian cells The antimicrobial effect of biosurfactants is manifested through detergent-like activities	[15,26,189,207]
	Anticancer activity	Sophorolipids	Biosurfactants as an antiviral agent, halt cell replication in favour of cell differentiation	[10,21]
	Immunological adjuvants	Surfactin,	Immunomodulating biosurfactants stimulate the immune system by increasing the ratio of lymphocyte transformation and migration of polymorph nuclear cells	[22,208]
	Antiviral activity	Sophorolipid diacetate ethyl ester, surfactin	Inactivation of viral lipid envelopes and capsid	[10]
	Anti-adhesive agents	Sophorolipids	Biosurfactants adsorption to a substratum modifies the surface hydrophobicity thereby interfering with microbial adhesion and desorption process	[149]
Bioprocessing	Product recovery	Sophorolipids Rhamnolipids	Biosurfactants form part of the reverse micelle extraction of antibiotics and proteins using their surfactant properties	[17,179,209]
Leather		Biodispersan	Degreasing: used as skin detergent, emulsifier; tanning and dyeing: wetting and penetration, and promoter	[175]
Textile		Trehaosetetraester Unspecified cHAL2	Removal of lipophilic components from fibre surface as a pre-treatment, removal of oil from fibres and enhanced dispersion of dyes for uniform and better penetration into fibre	[175]
Paper	Pulp processing	Biodispersan	Used for washing and deresinification of pulp by defoaming, dispersion and colour levelling	[175]
	Papermaking	Biodispersan	Limestone was effectively grounded using biodispersan and used as a filter in papermaking. Biosurfactant also used in calendaring through wetting, levelling, coating and colouring	[175]
Paint/coating protection		Biodispersan	Employed as a dispersant and as a wetting agent during grinding and stabilization for improved mixing property	[184]
Food industry	Food emulsifier	Polymeric biosurfactants	Modification of the rheological characteristics of the food to a desired consistency and texture using emulsification properties	[210]
	Food stabilizer	Rhamnolipids	Modification of the rheological characteristics of the food to a desired consistency and texture	[210]
Cosmetic industry		Sophorolipids Rhamnolipids MELs	Application of biosurfactants in cosmetics is due to their low irritancy, cytoprotective effect, anti-ageing, acts like an antioxidant, wettability, moisturizing properties, healing and skin toning features	[7,171,211]
Laundry detergents		Sophorolipids MEL	Properties such as foaming, surface tension reduction, solubilization make it suitable for detergent making	[172]
